# ‘Guidance' or ‘Misleading'? The government subsidy and the choice of enterprise innovation strategy

**DOI:** 10.3389/fpsyg.2022.1005563

**Published:** 2022-09-09

**Authors:** Jian Ding, Jiaxin Wang, Baoliu Liu, Lin Peng

**Affiliations:** ^1^Faculty of Business and Economics, University of Malaya, Kuala Lumpur, Malaysia; ^2^School of Accounting, Zhongnan University of Economics and Law, Wuhan, China; ^3^School of Economics and Management, Beijing University of Technology, Beijing, China; ^4^Discipline of International Business, Business School, The University of Sydney, Sydney, NSW, Australia

**Keywords:** innovation strategy, government subsidy efficiency, evolutionary game, dynamic strategy, high-quality industrial development

## Abstract

Government subsidies have a direct impact on firms' innovation strategies. The game relationship between the government, the subsidized firm and its competitors under different subsidy strategies affects firms' innovation behavior and thus innovation performance. This paper uses a dynamic evolutionary game theory approach based on cost-benefit differences to analyse the mechanisms by which government subsidy strategies affect firms' innovation strategies. It is found that the marginal benefits of a firm's innovation strategy will directly affect the game outcome, indicating that the choice of innovation strategy depends on the maximization of individual firm's interests. At the same time, a firm's innovation strategy is influenced by the firm's own innovation ability and competitors' innovation strategy, and there are two game equilibria. Government subsidies have a positive contribution to the innovation strategy choice of subsidized firms, but have a crowding-out effect on non-subsidized competing firms. The strength of the penalty (the efficiency of the implementation of government subsidies), the marginal revenue of the subsidized firms' rational use of government subsidies and the competitors' strategic choices will directly affect the game outcome.

## Introduction

Due to government subsidies have a positive impact on firms' innovation performance, market participants who receive government subsidies will produce more profits and greater social welfare (Sun H. et al., 2019; Guo et al., 2021; Li et al., 2022). The dynamic game played by the government, the firm, and its competitors determines the extent to which subsidy policy influences firm innovation performance (Clò et al., 2020; Shinkle et al., 2021; Ling et al., 2022). As a result, the interaction between the effectiveness of government aid and firms' capacity for learning and absorption ultimately determines the game's outcome (Carayannis et al., 2006; Mehmanpazir et al., 2022; Rodríguez et al., 2022). Despite government subsidies, firm heterogeneity allows businesses with varying production capacities to pursue a variety of innovation strategies (Lanahan et al., 2021; Kleine et al., 2022). Government subsidy programs frequently have a significant impact on technologically innovative businesses (Duan et al., 2022). Businesses' lack of innovation can render government subsidies ineffective (McDonald et al., 2021). Therefore, government subsidies and business innovation strategies have a game-like relationship that influences both the effectiveness of government subsidies and the effectiveness of business innovation.

Enterprises are one of the most active microeconomic carriers and platforms for innovation, making them one of the most important drivers of high-quality national economic development (Greenstein, 2010; Aistleitner et al., 2021). The performance of enterprise innovation will have a direct impact on the performance of industry innovation (Li X. et al., 2021; Dong et al., 2022; Yin and Yu, 2022). Consequently, businesses must take the initiative in industrial upgrading. Subsidies from the government have an impact on the market's ability to allocate resources decisively, and they are an important regulatory tool for directing high-quality economic growth (Wang et al., 2019; Lian et al., 2022). Innovation subsidy policies are effective at encouraging enterprise-level innovation and lowering the cost of innovation for businesses (Wu et al., 2022; Zhang et al., 2022). However, information asymmetry causes deviations in the implementation of government subsidy policies, resulting in a disparity between the effect of government subsidies as they are actually used and expectations (Nishimura and Okamuro, 2018; Bianchini et al., 2019). Both domestic and international scholars have conducted extensive research on government subsidies. The current research on how government subsidies affect firms' innovation performance is divided into two categories: boosting effect and inhibiting effect.

Innovation-driven development is a key strategy for China's current high-quality economic development, which is critical for China to achieve from catching up to surpassing and for China to create an innovative nation. As a result, the following areas are where this paper may add to existing research: First, explain how government policy on subsidies influences how well businesses innovate. Second, to investigate the effectiveness of government subsidy policy implementation on the performance of companies and industries in terms of innovation. Third, provide suggestions for improving the effectiveness of government subsidies in encouraging businesses to perform better in terms of innovation. This paper abstracts government subsidy policies and firm innovation performance into a game problem and investigates the mechanism of the role of government subsidy policies on firm innovation performance through an evolutionary game analysis of the game equilibrium point between government subsidy strategies and firm innovation activities.

## Literature review

The original goal of government subsidy policies is to promote business development and improve business performance; however, deviations in policy implementation and execution frequently result in policy misalignment and thus have multiple effects; and currently, the following are the main findings of government subsidy research.

### Positive effects

The primary feature of government subsidies is that they are not remunerative (Schwanitz et al., 2014; Ginn and Pourroy, 2022). Government subsidies are acts of financial expenditure in which the government allocates funds to businesses or individuals without compensation in order to achieve political, economic, and social goals (Bǎzǎvan, 2019; Habich-Sobiegalla and Rousseau, 2020). For a long time, government incentives were thought to encourage business innovation. The issues of high upfront investment, uncertainty regarding innovation output, and externality of innovation benefits have stifled enterprises' enthusiasm and initiative to carry out innovation activities (Santen and Anadon, 2016; Andries and Hünermund, 2020; Liu et al., 2022). Some of the risks associated with innovation activities can be shared with government funding (Roh et al., 2021; Yu et al., 2022). With the implementation of the innovative country strategy, government funding for science and technology innovation has increased, as have business innovation subsidies (Guo et al., 2022; Liang et al., 2022). Government subsidies can help businesses that rely on technology reduce the costs and risks of technological innovation, close the return on investment gap between society and businesses, and relieve financial pressure on these businesses (Li Q. et al., 2021; Bertello et al., 2022; Lenderink et al., 2022; Song et al., 2022). Hence, businesses are encouraged and motivated to innovate more frequently. It not only provides logical cash benefits to businesses, but it also sends messages (Al-Mamary and Alshallaqi, 2022; Fernhaber and Zou, 2022; Mai et al., 2022). Government-subsidized businesses typically have high technological content, strong overall strength, favorable development prospects, and so on (Meath et al., 2016). This may improve a company's ability to attract investors and raise public awareness of its market position.

There are two main ways in which government subsidies for business innovation are beneficial. Government subsidies, on the one hand, encourage businesses to increase their investment in technological innovation while lowering the cost of technological innovation (Pan et al., 2021; Acheampong et al., 2022; Wang and Zheng, 2022; Zahoor et al., 2022). Due to the significant sunk costs associated with innovation and the unpredictability of the results, some businesses choose not to pursue it (Vértesy, 2017; Mezzanotti and Simcoe, 2019; Lin et al., 2021). While government subsidies can help lower the sunk cost of the initial stage of technological innovation and encourage enterprises to carry out innovation activities, some businesses with the capacity to engage in technological innovation will also reduce their own innovation investment in light of the positive externalities and spillover effects of innovation results (Arza and López, 2021; Liu Q. et al., 2021; Fan et al., 2022; Jiang and Liu, 2022). Government subsidies, on the other hand, have a “signaling” function that allows businesses to get funding for innovation from more sources, increasing their capacity for technological innovation (Gao et al., 2021; Trotter and Brophy, 2022; Weiss and Nemeczek, 2022). Due to the insufficient financial market system and information asymmetry, bank credit primarily favors higher-paying applied research projects, while some high-risk basic research projects are difficult to finance (Kou et al., 2020; Cao et al., 2021). Government support for science and technology strengthens firms' capacity for innovation, acts as a “signaling” mechanism, broadens firms' funding sources, and reduces the cost of financing R&D projects, all of which increases the likelihood that some creative SMEs will be able to secure credit financing (Galbreath et al., 2021; Prokop et al., 2022; Roh et al., 2022). Furthermore, government funding for specific innovation projects may indicate to society the future demand for goods produced by the public sector (Lybæk et al., 2021). When this demand is combined with business demand for new products and services, the expected marginal rate of return rises, encouraging more businesses to engage in innovation activities.

### Negative effects

Appropriate government subsidies can help firms to innovate technologically, but excessive or too much subsidies may stifle technological innovation (Prud'homme et al., 2018; Ma and Li, 2021; Xie et al., 2022). For example, government subsidies for strategic emerging industries currently have a negative incentive effect on firm performance in China (Li X.-L. et al., 2021; Wenqi et al., 2022; Zuo and Lin, 2022). When an enterprise's subsidy income to sales ratio is less than a critical value, the subsidy can significantly improve productivity (Qiao and Fei, 2022); however, as the subsidy income ratio gradually rises to this critical value, the increase becomes insignificant, and a negative effect on enterprise productivity variation emerges (Ge et al., 2018; Bianchi et al., 2019). As the income-to-sales ratio rises and approaches a critical level, government subsidies impede company productivity growth (Szczygielski et al., 2017; Li et al., 2020; Wu Y. et al., 2020). Government subsidies have a substantial crowding out effect on firm R&D investment behavior, and the amount of R&D subsidies increases innovation costs while limiting production efficiency advances (Ludkovski and Sircar, 2016; Carboni, 2017). Therefore, sponsored enterprises may outperform unsubsidized companies while being less efficient in meeting program objectives such as labor productivity and value creation (Hottenrott and Richstein, 2020). As a result, while direct government subsidies benefit enterprises in the short term, they may inevitably impede their long-term growth (Luo et al., 2021; Yan et al., 2022). Due to the two-fold spillover problem associated with innovation, companies distribute subsidies differently between non-innovation and innovation, with the favorable effect of subsidies on non-green innovation being bigger than the good effect on innovation (Frankovic et al., 2020; Banal-Estañol et al., 2022; Huang et al., 2022). Furthermore, non-tax subsidies are used by the government to advance social policy goals at the expense of commercial profits.

Government subsidies, on the other hand, have a substantial positive link with a decrease in profit management (Ma, 2022; Sun et al., 2022). When bad corporations gain subsidies because of their political affiliations, they drive out excellent companies that do not receive subsidies, eroding profit margins (Patnaik et al., 2022). Therefore, government subsidies have a negative moderating influence on entrepreneurial growth. Although political connections influence the quantity of invention, they may have a negative impact on its quality (Liu S. et al., 2021; Wang et al., 2022). Political ties hinder government's ability to fund high-quality innovation subsidies and even lower industry R&D intensity, a key indicator of innovation quality (Tian et al., 2019; Yao et al., 2022). Government subsidies, although encouraging independent innovation in high-tech industries, have a negative impact on company performance in both low-tech and high-tech companies (Wu and Hu, 2020; Guo and Zhang, 2022). According to several studies, knowledge asymmetry between government and business is a major cause of squandered subsidies (Chen et al., 2020; Khan and Krishnan, 2021; Krukowski and DeTienne, 2021; Mas and Gómez, 2021; Kohlbrecher and Merkl, 2022). The foundation of this reaction is based on imprecise information transmitted between the government and companies in a game involving subsidies and innovation strategy, with the game's outcomes directly influencing the business's decision on innovation strategy (Ling et al., 2022; Song et al., 2022; Zhang and Yu, 2022).

Overall, there is no clear positive or negative influence of government subsidies on corporate innovation. In reality, there is a U-shaped link between government subsidies and the efficiency of innovation (Liu et al., 2019; Ahn et al., 2020; Xia et al., 2022). Reduced government subsidies prior to the tipping point can enhance innovation efficiency, and the incentive impacts of R&D subsidies vary greatly depending on funding limitations (Sun X. et al., 2019; Björkegren and Karaca, 2022). To correct market flaws associated with business R&D operations, government action is required in a visible time frame (Yasir et al., 2020). Subsidies lower the amount of market capital required by creative companies, lowering the cost of capital (Wu T. et al., 2020). Companies that receive subsidies concurrently provide an information signal to market-based investors, making it easier for them to raise capital.

In conclusion, while existing literature has already examined the interaction mechanism between government subsidy programs and corporate innovation performance to some extent, few have definitively proven the link. As a result, the focus of this paper is on the interactions between government subsidies and entrepreneurial innovation. An evolutionary game model is used to explore their interaction in order to understand how firms might accomplish efficient innovation when government subsidies and rivals' strategy choices are combined.

The objectives and contributions of this paper are threefold. First, this paper investigate the mechanism of interaction between government subsidies, innovation capability, competitor strategy, and firm innovation performance, beginning with the most important factors influencing firm innovation, such as government subsidies, innovation capability, and competitor strategy. Second, when it comes to research approach selection, the dynamic game model may provide a more accurate picture of the alternatives. Finally, based on relevant studies regarding the impact of government subsidies on innovation performance, we focus on the impact of competitive strategy choice and government subsidy efficiency on corporate innovation.

## Research design

### Assumptions of the game model

#### Participants in the game

According to Sung (2019) and Chen et al. (2019), the three players in the game are the fiscal subsidies provided by the government, subsidized enterprises, and unsubsidized firms in the relevant subsidized industries. The three parties in the game are constrained rationality. Firms will spend in creative activities, mostly R&D and internal system reform in order to obtain competitive advantages and maximize profits in an imperfectly competitive market. And these inventive efforts, will then be transformed into real revenues and benefits. Few firms would actively engage in innovation activities due to the high level of unpredictability and significant investment necessary in corporate innovation operations. Subsidies from the government have become an essential component in influencing enterprises' innovation activities, and they have the ability to dramatically increase companies' passion for innovation.

However, two issues arise as a result of the government's and companies' lack of information. (1) The pressure of innovation debt causes certain supported firms to use government subsidies for other reasons due to the high unpredictability of the benefits given by innovation. (2) In order to qualify for government subsidies, some firms establish innovation projects or separate innovation operations, which can result in moral hazard, uneven resource allocation, and poor innovation performance.

#### Behavioral strategies of game participants

In imperfectly competitive marketplaces, firms compete largely on output and price advantages, with the latter being more relevant. As a result, a firm's market competitiveness is frequently linked to its innovative initiatives. The Bertrand model is used to test the following hypotheses to better understand the relationship between the efficiency of government subsidy policies implemented by firms and their innovation performance, as well as to rule out the impact of competitive market pressures on firms' innovation activities.

*Hypothesis 1*: On the market, there are only two companies: *FirmA* and *FirmB*. The products of *FirmA* and *FirmB* are highly interchangeable, and *FirmA* owns the technology to manufacture the product at a cost of *C*. Anyone who wants to enter the market can do so, and firms compete mostly on price. Customers will only buy the product from the lower-priced company, so: MaxB_*i*_(*p*_*i*_, *p*_*j*_) = (*p*_*i*_−*C*) × *N*_*i*_(*p*_*i*_, *p*_*j*_). In the formula *C* is the cost, *N* is the market demand, customers will only buy the product from the lower-priced company. So:


(1) $$N_{i}(p_{i},p_{j})=\{\begin{array}{l} N(p_{i}^{\;}),p_{i}<p_{j}^{\;}\\ 1/2\;N(p_{i}),p_{i}=p_{j}\\ 0,p_{i}>p_{j} \end{array}$$

For firms to innovate, there are two basic approaches: They can begin by innovating from within, such as mastering key technologies through autonomous invention and gaining a unique competitive advantage accordingly. The second option is to engage in stream innovation, which means combining current technology with significant foreign differences by incorporating foreign ready-made technology and integrating existing resources for integrated innovation. Companies innovate for three primary reasons: First, create new products to set market trends, and becoming the market leader and dominant player ultimately; second, gain a competitive price advantage in the market by lowering existing production and operating costs, i.e. process innovation; and third, maintain market sensitivity, i.e. the ability to absorb and digest external information (responsiveness). Therefore, the major purpose of corporate innovation is to minimize existing production and operational expenses. Because of the differences of corporate innovation capabilities in different firms, the choice of enterprise innovation strategy, as well as the performance of innovation, will fluctuate. *FirmA*'s innovation capability is influenced by elements such as strategic planning capability, marketing capability, learning capability, R&D capability, and others. High levels of competency in other fields translate into high levels of innovative performance. As a result, investigate the costs and benefits of innovation in order to demonstrate discrepancies in innovation activities among organizations with various innovation capabilities, and generate hypotheses:

*Hypotheses2*: If *FirmA* is willing and able to innovate, similar foreign enterprises' production and operation technology can reduce the existing production and operation costs to *M-N* (*M* is the original cost, *N* is the value of the reduction in operation costs caused by the introduction of a management mode or technology), but the foreign enterprises will monopolize and block the existing production and operation technology. To maintain their market position, foreign firms will monopolize their existing production and operation technologies, and will only transfer their old production and operation technologies to domestic firms at a price of “*Z*” if they have more advanced production and operation technologies, or if domestic *FirmB* independent innovation threatens their position.

*Hypotheses3*: If *FirmA* has become the leader in the domain through its own innovation, the cost of production and operation will fall dramatically, and as the scope and depth of its innovation activities expand, the cost of production and operation will fall to a very low level in the industry, which is assumed to be zero for intuitive purposes. In this situation, the foreign firm may provide its technology at a reduced cost to *FirmB* in order to stimulate competition between the two firms and limit *FirmA*'s expansion in order to retain market dominance or monopoly.

*Hypotheses4*: The cost of innovative activity at *FirmA* is *I*. When *I* > *Z*, the cost *I* is a sunk cost, and the firm's ability to innovate determines the outcome of its innovation attempt. If, on the other hand, is not as innovative, it will have to pay higher costs *(I*+*q)* to attain the desired result. Due to a lower level of innovation, competitor *FirmB* may have more options in the two scenarios, resulting in a cost difference of *2q*.

### Construction of the model

Based on the above research, we may deduce whether a corporation regards innovation as a game: Enterprise innovation aims to reduce existing production costs, but its ability to do so is restricted by a shortage of resources. Therefore, enterprise innovation mode selection should be based on both the enterprise's own innovation capacity and the ability of market rivals to innovate independently, as well as the use of flexible selection based on strategy in connection to revenue maximization. Although competitors will be able to accurately forecast the outcome of enterprise innovation due to the enterprise's highly uncertain innovation activities, resulting in an imperfect information dynamic game theory, competitors will not be able to accurately forecast the outcome of enterprise innovation due to the enterprise's highly uncertain innovation activities.

Because information spillovers from innovative activities are rather gradual, foreign firms would eventually lose their advantage due to the knowledge spillover effect, while local organizations will be able to access important production and operation technology at a low cost. If the foreign company transfers the relevant production and operation technology to the domestic company before the enterprise's independent innovation is successful, the knowledge spillover from the innovation will be accelerated, and even if the domestic enterprise carrying out independent innovation has not yet acquired the advanced production and operation technology, it will be able to master it quickly through the knowledge spillover from the innovation. Enterprises that have not engaged in innovation activities or have a low capacity for innovation, on the other hand, are unable to absorb the benefits of the spillover effect quickly because they are lacking the necessary foundation and can only acquire the necessary technology at a high cost of technology transfer. Simultaneously, the spillover effect of innovation allows imitators to get ideas at a lower cost, resulting in firms that innovate earning lower returns than society, resulting in “inertia” in innovation. Inertia restricts a company's ability to develop and enhance its inventive capabilities. Financial subsidies are increasingly being used by governments to mitigate the negative impacts of innovation spillovers.

Simultaneously, there are two primary behavioral strategies for subsidized enterprises' use of government subsidies: leveraging the subsidies for creative inputs into government-supported projects or using the subsidies for non-project-related commercial operations to profit. In this context, “using government subsidies for innovation” refers to companies that use government subsidies for entrepreneurship, technological advancement, and innovation in industries targeted by the subsidy policy, with the goal of increasing the rate of results transformation and promoting the development of subsidized industries through increased business innovation capacity. The term “use of government subsidies for other profit-making activities” refers to the use of government subsidies by subsidized enterprises for profit-making activities unrelated to the development of the supported industry.

Meanwhile, the government must monitor how subsidized companies use government subsidies, and it has two behavioral strategies: monitoring and checking whether companies use subsidies to develop innovative activities, or not monitoring and checking whether companies use subsidies to develop innovative activities. As a result, whether a subsidized corporation uses government subsidies for innovative activities in the project defined by the subsidy and gains a competitive edge in the market can be viewed as the result of a game played between the government and the firm. The following additional assumptions are created as a result of the prior study and hypothesis 4:

*Hypothesis5*: As a result of the government's numerous monitoring mechanisms for how supported firms use the subsidies, the following requirements exist: ①When a firm is strong in innovation, it will innovate no matter whether the government is watching or not, lowering the firm's innovation cost to (*I-q-S*) at this stage, where “S” represents the amount of subsidies received. ②When the firm's potential for innovation is insufficient, the government's monitoring approach determines whether the firm innovates and whether the subsidy is used to support the stated project's innovation activities. Because the government's monitoring approach affects the firm, there are three choices for whether it will invest in innovation: whether it is monitored or not, its cost is (*I*+*q-S*), and if it is not monitored, it does not invest and its innovation is zero. The cost is (*I*+*q*+*S*+*f* ) because it is monitored but not invested, with f denoting the penalty to the firm if it is discovered that the subsidy was not used to invest in the firm's creative operations. Under this situation, therefore, FirmB is in a terrific position to participate in the event.

**Figure 1:**
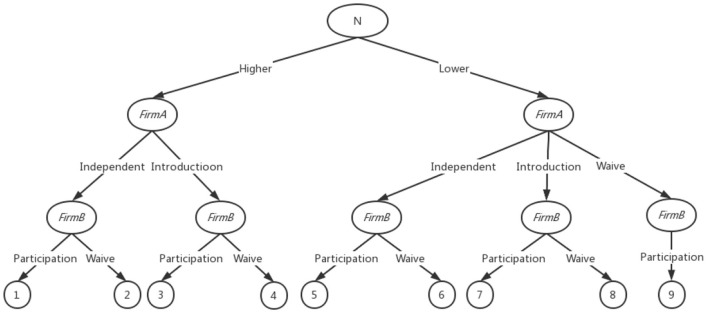
An extended representation of a firm's innovation activities.

As a consequence of prior analysis, a game model that permits companies to engage in innovative activities has been developed and merged with hypothetical settings in which the subsidized *FirmA* has first-mover advantage. The first stage is to assess *FirmA*'s level of innovation capability. The probability of high innovation capability is β, and the probability of low innovation capability is *(1-*β*)*; *FirmA*'s second stage is to understand its innovation capability and choose an innovation strategy to reduce costs and increase revenue; competitor *FirmB*'s third stage is to learn about *FirmA*'s innovation strategy but not know whether *FirmA* has a high or low innovation capability, and then decide whether to innovate. The third stage happens when competitor *FirmB* learns about *FirmA*'s innovation plan but is skeptical of *FirmA*'s ability to develop. *FirmB* then determines whether to invest or not. Figure 1 depicts the evolutionary game formulation with limited information.

If subsidized *FirmA* innovates on its own and achieves remarkable success, its production costs fall to the lowest in the market because its advanced production operation model outperforms other peer firms at home and abroad, at which point *FirmA* enjoys the entire market revenue *R*, and *B(0)* = *1/4*. If *FirmA* launches a new product while competitor *FirmB* remains silent, *FirmA* retains exclusive market access, and its benefit is B_1_(M-N) = [1-M+N]/4. If a competitive *FirmB* enters the market and both share the market proceeds, the proceeds are B_2_(M-N) = [1-M+N]/8. If *FirmA* does not invest in new activities due to a lack of innovation capabilities, competitor *FirmB* gains exclusive market access by bringing advanced foreign manufacturing and operation technologies, resulting in *FirmB* having a competitive advantage (>0). The current return on *FirmA* is zero. The extended equation can be used to compute the return functions for *FirmA* and *FirmB* (Table 1). And *B*(0)>*B*_1_(*M*−*N*)>*B*_2_(*M*−*N*)>0.

**Table 1:** Participant's return function.

**Number**	**Revenue matrix**
1	(B(0)-(I-q-S), -Z)
2	(B(0)-(I-q-S), 0)
3	(B2(M-N)-Z,B2(M-N)-Z)
4	(B1(M)-Z, 0)
5	(-(I+q-s), B1(M-N)-Z)
6	(B(0)-(I+q-S), 0)
7	(B2(M-N)-Z, B2(M-N)-Z)
8	(B1(M)-Z, 0)
9	(0, θ)

### Model analysis

Because of the influence of government subsidy policy, when competitor *FirmA* succeeds in independent innovation with high independent innovation capacity, *FirmA*'s production and operation technology is better than similar foreign enterprises at this time, and the production cost drops to the industry's lowest standard, *FirmB*, even if it introduces advanced foreign production and operation technology, as long as the enterprise sets the product price at *M-N-*α. In other words, as long as *B2(M-N)-Z* ≧ *0, FirmB* can import foreign manufacturing and operation technology to compete with the firm in the remaining situations. This also proves that *Hypothesis1* is correct.

On the other hand, when *B*(0)−*B*_1_(*M*−*N*)>*I*−*q*−*S*−*Z*, foreign import innovation has far fewer advantages than self-invention. *FirmB* will compete with sponsored *FirmA* in the third stage regardless of whether subsidized *FirmA* invests in creative activities, and will sell its products at a lower price than *FirmA*, removing *FirmA*'s competitive edge. The market share of subsidized *FirmA* will be taken, and *FirmA* will be forced out of the market. When under competitive pressure, subsidized firms with limited innovation capacity would typically use subsidies to innovate (launch inventions), increasing the ability to compete with rival firms, even though the competing firm's optimal strategy is to introduce first and launch first to get market access. If, on the other hand, the subsidized firm is extraordinarily innovative, it will develop on its own regardless of the competitive threat, and the presence of the subsidy will considerably increase its risk tolerance for innovation. Rival companies, on the other hand, will not compete in the market to boost their own earnings. In practice, government subsidies have a horse-trading effect (Ding et al., 2021), indicating that they may have “sticky effect” that leads to a “winner-takes-all” situation (Hsiao et al., 2016). Subsidized enterprises with high innovation capacity will choose to innovate independently for their own benefit, whereas subsidized enterprises with low innovation capacity will opt for the introduction strategy, and all rival enterprises will compete as long as the subsidized enterprises opt for the introduction strategy.

When *B*(0)−*B*_2_(*M*−*N*) < *I*−*q*−*S*−*Z*, because the costs of autonomous innovation are so high, the benefits are smaller than those acquired from imported innovation, and even lower than those derived from market competitors. It follows that *B*(0)−(*I*−*q*−*S*) < *B*_2_(*M*−*N*)−*Z*<*B*_1_(*M*−*N*)−*Z*, Regardless of its innovation potential, the subsidized enterprise should choose to embark on an innovation plan now. Because the subsidized firm will be unable to develop on its own at this time, the competitor will profit as long as it participates in the market, thus the competitor firm will compete in the market regardless. At this stage, the sponsored firm foregoes independent innovation, allowing the rival firm to compete in the market.

When *B*_2_(*M*−*N*)−*Z*<*B*(0)−(*I*−*q*−*S*) < *B*_1_(*M*−*N*)−*Z*, Subsidized enterprises with low innovation capacity do not choose to innovate on their own at this time, while firms with high innovation capacity can innovate on their own or introduce innovation at random, and market competitors can compete at random. Let ***x*** represent the possibility of a firm with strong innovation capabilities engaging in autonomous innovation and ***y*** represent the likelihood of a competitor engaging in market competitiveness. The expected returns to both are:

The expected return of the subsidized *FirmA* is (*Q*), then we have:


(2) $$\begin{array}{l} Q\text{ = }xy\text{[}B\text{(0)}-\text{(}I-q-S\text{)] + }x\text{(1}-y\text{)[}B\text{(0)}\\ \;-\text{(}I-q-S\text{)] + }y\text{(1}-x\text{)}\\ \;[B_{2}(M-N)-Z]+(1-x)(1-y)[B_{1}(M-N)-Z] \end{array}$$

When the benefits of subsidized *FirmA* are maximized, the $\frac{\partial Q}{\partial x}=0$, It can be concluded that:


(3) $$y=\frac{B_{1}(M-N)-Z-[B(0)-(I-q-S)]}{B_{1}(M-N)-B_{2}(M-N)}$$

The expected return for competitor *FirmB* is (*M*), then:


(4) $$\begin{array}{l} M=\;\beta xy(-Z)+\beta (1-x)y[B_{2}(M-N)-Z]\\ \;+(1-\beta )y[B_{2}(M-N)-Z] \end{array}$$

When the interests of competitor *FirmB* are maximized, the $\frac{\partial M}{\partial y}=0$, It can be concluded that:


(5) $$x=\frac{B_{2}(M-N)-Z}{\beta B_{2}(M-N)}=\frac{1}{\beta }(1-\frac{Z}{B_{2}(M-N)})$$

The equilibrium point at this point [x^*^, y^*^] is:


(6) $$\begin{array}{l} \{\frac{\text{1}}{\beta }(1-\frac{Z}{B_{2}(M-N)}),\\ \frac{B_{1}(M-N)-Z-[B(0)-(I-q-S)]}{B_{1}(M-N)-B_{2}(M-N)}\;\} \end{array}$$

When *y*>y^*^, subsidized *FirmA* chooses an autonomous innovation strategy, When *y*<y^*^, Choose to introduce innovative strategies. And when *x*>x^*^, competing firm b has chosen not to compete in the market, But *x*<x^*^, then compete in the market.

According to the findings, the chance of competitor FirmB participating in the market are adversely connected to subsidized *FirmA*'s innovation ability and positively related to the benefits gained by competing in the market. If the subsidized firm's innovation capacity is higher, i.e. the higher β, the result is a smaller x^*^, the greater the probability that *x*>x^*^, The less likely a competitor is to compete in the market. When B2(M–N) is larger, the greater the probability that *x*<x^*^, the greater the gain to firm b, the greater the probability of entering the market to compete. As a result, the chance of a corporation engaging in autonomous innovation is positively related to the amount of the monopolistic benefits it can get and negatively related to the gains it can achieve by pursuing an introduced innovation strategy.

### Efficiency of government subsidy policy implementation and innovative strategy options

According to prior research, the use of government subsidies by subsidized enterprises is the result of a game between the government and rivals based on their own innovation potential under the government subsidy policy. Subsidized firms with low innovation capacity rarely choose the path of independent innovation due to the temptation of introduction and the threat of competitors, whereas firms with high innovation capacity face the same problem and are unsure whether to pursue independent or introduced innovation strategies.

If the probability of a firm undertaking an innovation receiving a government subsidy is δ , then the cost to the firm of undertaking the innovation is (*I*±*q*) × (1−δ). Consequently, the advantages of autonomous innovation have altered. When a competitor's odds of competing in the market remain the same, the government subsidy raises the firm's gains from autonomous innovation, and the firm chooses to invest in it. However, because the government has the authority to monitor how corporations use subsidies, if a subsidized firm uses the subsidy for innovative activities, the subsidy will be revoked, and the cost of innovation will be (*I* ± *q*−*S*) × (1−δ). The government detects and penalizes the firm since it has a low capacity for innovation and uses subsidies for sectors that maximize its own interests rather than new inputs, therefore the cost of innovation is (*I*+*q*+*S*+*f*) × (1−δ). The government subsidies are currently crowding out the company's creative efforts. There is a crowding-out effect when the subsidy is applied, and because the government is in charge, there is a rent-seeking effect.

Because the subsidized firms' innovation strategy is undetermined, set λ = 1 implies that they choose their own innovation strategy, set λ = 0 denotes that they choose to introduce innovation, and set λ = -1 denotes that they do not invest in innovation. As a result, the subsidized firms' winning function under government subsidy policy is:


(7) $$\begin{array}{l} F(M|S)=\text{λ}B(0)+(1-\text{λ})B_{2}(M-N)-\text{λ}(I\pm q-S)\\ \;(1-\delta )-(1-\text{λ})Z\;(\text{λ}\in \{-1,0,1\} \end{array}$$

Despite the government's agreement to fund innovation efforts, it is crucial to identify the size and proportion of the subsidy, as well as the post-subsidy monitoring approach, because these elements will have a substantial impact on the innovation performance of subsidized firms. So that λ = 1 = *Max*_λ_*F*(*M*|*S*), which requires the following:


(8) $$\begin{array}{l} \frac{\partial F(M|S)}{\partial \text{λ}}=B(0)-B_{2}(M-N)-(I\pm q-S)\\ \;(1-\text{λ})+Z\geq \text{0} \end{array}$$

which gives *B*(0)−(*I*±*q*−*S*)(1−λ)≥*B*_2_(*M*−*N*)−*Z* (9)

Government subsidies should be sufficient to allow subsidized companies to expect returns on their own innovation that are equivalent to or greater than those expected on fresh invention. That is


(10) $$\delta \geq \frac{B(0)-(I\pm q-s)-[B_{2}(M-N)-Z]}{I\pm q-S}$$

Consequently, when government subsidies are insufficient or poorly implemented, the expected goal of improving enterprise innovation performance is not met, and companies will only innovate on their own when subsidies reach a certain level; otherwise, they will prefer to introduce innovation (flow innovation), making it difficult to understand the industry's pioneering power in international trade.

Although government subsidies are effective at increasing expected income and decreasing risk aversion among firms, the objective reality is that when competitors decide to enter the market and compete, changes in the revenue structure and actions of subsidized firms will influence their business plans and development of new competitive strategies. Because of the existence of governmental monitoring and punishment mechanisms, firms with low innovation capacity will continue to prefer to implement strategies to innovate rather than use subsidies in other areas that maximize their own returns, whereas firms with high innovation capacity and market competitors' equilibrium strategies are:


(11) $$\begin{array}{l} \{x^{{*}^{\prime }},y^{{*}^{\prime }}\}=\{\frac{1}{\beta }(1-\frac{Z}{B_{2}(M-N)}),\\ \frac{B_{1}(M-N)-Z-[(B(0)-(I-q-S)(1-\delta )]}{B_{1}(M-N)-B_{2}(M-N)}\} \end{array}$$

Consequently, when the government subsidizes enterprise innovation, the likelihood of competitors competing in the market decreases as the strength of government subsidies increases, i.e., the strength of government subsidies is negatively related to the probability of competitors competing in the market, but enterprises remain unaffected as the primary source of innovation. This helps to explain why, despite government subsidies, certain industries continue to rely largely on imports for innovation, resulting in lower independent innovation performance. The consequences of crowding out and rent-seeking limit innovation even further.

According to the analysis above, the efficiency with which government subsidy policies are implemented (the level of monitoring) has a significant impact on whether firms use government subsidies to maximize their own returns or to engage in innovative activities, whereas the level of implementation efficiency is influenced by the level of penalties, resulting in subsidized firms' returns in areas where they maximize their own returns being lower than the returns obtained by firms that do not use government subsidies. Because policy implementation efficiency is dynamic, and the final equilibrium is defined by both sides' dynamic capacities, no evolutionary stabilizing mechanism (mainly learning skills) exists in this situation. Therefore, whether companies choose to engage in innovative activities with government subsidies or not is influenced by the government's subsidy policy plan, with the government's goal being to invest and gain advantages. As a result, a favorable mutual trust relationship between government and companies reduces the cost of implementing government subsidy policies while simultaneously encouraging the growth of supported enterprises and industries.

Based on the assumption that both the government and subsidized firms are finitely rational, the study employs an evolutionary game technique to analyze the interaction mechanism between government subsidies, subsidized firms, and subsidized firms' competitors under government subsidy programs. The severity of the punishment for noncompliance with the subsidy (the effectiveness of the government subsidy policy), the marginal benefit of the subsidized firm's rational use of the government subsidy, and the competitor's strategy all influence the game's result.

## Research results and analysis

### Research result

The results of the aforementioned analysis of government subsidies and enterprises' choice of innovation strategies are summarized as follows. First, A company's own innovation capability is at the heart of its innovation strategy. The choice of an organization's innovation strategy determines its success. Government subsidies are merely a moderator, as the most important factor determining an organization's choice of innovation approach remains its own innovative capacity. If a corporation possesses considerable innovation skills, choosing to innovate on its own will improve its monopoly advantage, whereas choosing to introduce innovation will cut the cost of innovation even further, giving it a market advantage. When a firm's internal innovation capacity is restricted, government subsidies have a greater moderating effect on the choice of innovation approach. Government subsidies may encourage firms to invest in self-innovation, but they can also assist enterprises in lowering the cost of introducing new technology, thereby improving their innovation performance.

Government subsidies amplify the innovation gap. Companies with strong innovation capabilities will be able to expand their market competitive advantages as a result of government subsidies, while those with weak innovation capabilities will have their survival space reduced. Sponsored companies are less likely to produce independently as a result of government subsidies, increasing China's reliance on other countries in critical technology fields at the expense of independent innovation. Concurrently, a subsidy program may encourage firms to overinvest, lowering innovation performance and producing a cobra effect.

Government subsidies have a positive incentive effect on the creative behavior and operations of subsidized firms. Subsidies from the government have just a tiny positive incentive effect on corporate innovation. Companies are the major subjects of innovation in market operations, with the goal of establishing a monopoly or competitive advantage in the market as a result of the innovation. Price competition, quality competition (value competition), and service competition are the three primary reasons of monopoly or competitive advantage. Government subsidies encourage subsidized firms to invest in innovation, but the effectiveness with which subsidy programs are executed influences the quality of subsidized investment in innovation. Simultaneously, the difference in innovation costs between supported and non-subsidized companies has a crowding-out effect on non-subsidized competing enterprises that have limited or no investment in creative activities.

Government subsidies have a positive relationship with the innovation strategy choices of subsidized firms. Government subsidies have a positive incentive effect on the selection and performance of subsidized enterprises' innovation strategies, albeit this effect is influenced by the firms' competitors' innovation strategies as well as its own innovation potential. When a company's internal innovation capability is strong, its innovation strategy favors independent invention over collaborative innovation; whereas, competitors' plans favor collaborative innovation. The firm's innovation strategy encourages introduced innovation, and subsidy policy has a negative impact on the firm's capacity for innovation. The decisions of competitors' innovative strategies have just a little impact.

The effectiveness of government subsidy schemes is tied to the choice of innovation methods and the performance of supported enterprises in terms of innovation (regulatory efficiency). The success of government subsidy policies has a positive impact on enterprises' choice of innovation approach and is unaffected by their ability for innovation. Enterprises will invest in innovation rather than divert government subsidies to other uses if the government subsidy program is more efficient (regulatory efficiency), regardless of their ability to innovate.

### Countermeasures and suggestions

The following steps should be taken to increase the effectiveness of government subsidies, significantly increase the level of innovation used by businesses receiving government subsidies, and lessen the risk of fraudulent subsidy use and subsidy diversion. Improve the effectiveness of government subsidies and the performance of industrial innovation by optimizing the distribution of subsidy resources. Subsidized enterprises are the primary drivers of regional industrial and economic advancement since they are selected by the government after a multi-party evaluation and have a specific development potential. Industrial innovation is risky, and the risk and investment made by a single business with its own resources is significant. The government's subsidy system will not only reduce the cost and risk of company innovation, but it will also encourage social capital to enter the market, relieving pressure on enterprises to innovate.

Fair subsidies, on the other hand, should be applied in conformity with the Industrial Technology Development Law. On the one hand, the government should improve policy direction and strengthen the design of important market processes so that the income generated by subsidized projects exceeds the income generated by other types of subsidies. On the other hand, to improve the efficiency of policy implementation, heavier penalties and supervision, with the increase of fine *f* , subsidized enterprise subsidies will be used for any other purposes, the lower the marginal revenue, far more than its gains, in the process of long-term learning adjustment, subsidized enterprises will choose government subsidies for innovation activities, to ensure their maximum profit. During this procedure, the government can check subsidized firms on a regular basis to prevent them from exploiting government subsidies in ways that benefit their own interests. Simultaneously, it is critical to deepen subsidy policy reform, strengthen the subsidy project review and approval procedure, and encourage frontier technologies with common features and major economic and social benefits that aid industry development. More assistance should be provided to private firms, eastern and western enterprises, and non-high-tech organizations with considerable innovation capability but little innovation capital.

Diversifying government subsidies, boosting collaboration between firms, colleges, and research institutes, and diversifying government subsidies would all help to increase the rate of innovation conversion. Enterprises may misappropriate earlier fixed-amount government payments for other reasons, making it more difficult for government subsidies to fully exercise their effects; at the same time, the “sticky” effect of government subsidies would dampen other enterprises' enthusiasm for innovation. Therefore, the government should analyze the inventiveness of enterprises seeking subsidies and apply flexible subsidies to improve the efficiency with which subsidy programs are implemented. For projects with a long innovation cycle and in the planning period, pre-, during, and after-stage batches can be used, and for projects with a long innovation cycle and in the planning period, a fixed number of subsidies can be used ahead of time for scientific and reasonable evaluation of innovative projects that have been launched and have great prospects but are experiencing financial difficulties. Subsidies will be more conducive to improving subsidy efficiency. The amount of the subsidy is estimated for firms with sufficient funds based on the firm's actual innovation cost after a successful invention. Subsidies of this type are advantageous because they can encourage similar companies to be more creative. In addition to direct financial subsidies, interchange platforms between scientific research institutions and enterprises can be created to promote industry-university-research collaboration and innovation conversion rates.

Improve the subsidy supervision mechanism and develop a mutually beneficial government-business relationship. To combat the rent-seeking effect of government subsidies (Jiang et al., 2018), government should develop a comprehensive whole-process supervision system, as well as a thorough understanding of the actual use and true flow of subsidy funds, to ensure that subsidy funds are used entirely for superior R&D. Establishing a positive working relationship with subsidized firms based on mutual trust and reciprocity would not only boost corporate profits, but will also aid in the growth and development of supported industries. Strive for an evolutionary equilibrium in which the government opts for a non-verification policy and subsidized firms employ their subsidies in creative activity. This is a win-win situation for both the government and the companies. An evolutionary stability strategy will undoubtedly enhance the cultivation and development of supported industries.

## Discussion

The differences in firm innovation and firm innovation strategies between subsidized firms, the government, and competitors as a result of subsidy policies are investigated in this paper. The interaction between subsidized businesses, the government, and competitors is investigated. From a theoretical standpoint, the influence mechanism of government subsidies on firm innovation is examined, and the connection between government subsidy policy and the decision of firm innovation strategy is clarified.

### Theoretical contribution

This paper studies the formation process of government subsidy strategy on enterprise innovation strategy selection by developing a dynamic game model of enterprise innovation strategy induced by a change in government subsidy strategy. Furthermore, this paper compensates the shortcomings of previous studies, which are mostly reflected in the fact that the majority of previous studies are empirical studies based on quantitative data (Howell, 2017; Bai et al., 2019; Du and Li, 2019; Lin and Luan, 2020a,b; Huang et al., 2022). Empirical studies only offer data results, so they analyze existing views primarily through data and frequently lack a clear understanding of how objective patterns are formed. It is challenging to develop a thorough understanding of the actual impact mechanisms because such studies frequently only identify the impact of specific data indicators on firms. This research abstracts government subsidy policy and enterprise innovation strategy as a game problem, studies the mechanism of government subsidy policy on enterprise innovation, and makes relevant recommendations based on game theory.

### Practical contribution

The success of a company's innovation strategy is tied to the success of the company's innovation strategy. An organization's ability to innovate is a significant aspect in determining its innovation strategy. Government subsidies can only function as a brake. Because government subsidies can mitigate the negative impact of financing constraints on the performance of industry-university-research cooperative innovation to some extent, prior fixed-amount government subsidies may be embezzled by enterprises for other purposes, making it difficult for government subsidies to perform their functions. Therefore, government subsidies have a positive incentive effect on the choice of innovation strategy and innovation performance of subsidized enterprises, albeit this effect is modified by their competitors' choice of innovation strategy and their own innovation capabilities. Simultaneously, a single enterprise uses its own resources to carry out risk and investment in innovation, and the government's subsidy behavior will reduce the cost and risk of enterprise innovation while also guiding the entry of social capital and relieving pressure on enterprise innovation in the industry. Because the marginal advantage of supported firms embezzling subsidies for other purposes declines as fines increase, the benefits they receive far outweigh the costs. Hence, the government has theoretical basis for enhancing the efficiency of subsidy use supervision as a result of this study.

### Limitation and future research

The limitations of our study are threefold. First, with no data or empirical backing, this study simply suggests a game model and determines the game's equilibrium point. As a result, further data-driven study is required to support this hypothesis. Second, the study's proposal for the efficiency of government subsidies is fairly broad, and it is unclear if it refers to the efficiency of government subsidies in terms of oversight or the efficiency of enterprises that get subsidies. Finally, this article has certain limitations because it accepts that all government subsidies are direct money subsidies rather than other policy subsidies.

## Data availability statement

The original contributions presented in the study are included in the article/supplementary material, further inquiries can be directed to the corresponding author/s.

## Author contributions

JD: conceptualization, methodology, software, and writing—original draft preparation. JW: validation, formal analysis, investigation, resources, supervision, funding acquisition, and supervision. BL: data curation, writing—review and editing, and visualization. LP: writing—original draft preparation and data collection. All authors have read and agreed to the published version of the manuscript.

## Funding

The Ministry of Education's Humanities and Social Sciences Research Youth Fund Project Government Organization Reorganization and Enterprise Product Safety Information Disclosure-Quasi-Natural Experiment Research Based on the Newly Established Municipal Supervision Bureau (20YJC630143), National Natural Science Foundation of China Youth Project Stable Employment in the United States, Cross-border Migration of Science and Technology Talents and Innovation of Chinese Enterprises (72102229), and Hubei Province Soft Science Surface Project Research on the Technology Breakthrough of Hubei's Infrared Imaging Industry under the Crisis of Epidemic Core Shortage (CXRK2022000239).

## Conflict of interest

The authors declare that the research was conducted in the absence of any commercial or financial relationships that could be construed as a potential conflict of interest.

## Publisher's note

All claims expressed in this article are solely those of the authors and do not necessarily represent those of their affiliated organizations, or those of the publisher, the editors and the reviewers. Any product that may be evaluated in this article, or claim that may be made by its manufacturer, is not guaranteed or endorsed by the publisher.
